# Assessing Projection Bias in Consumers’ Food Preferences

**DOI:** 10.1371/journal.pone.0146308

**Published:** 2016-02-01

**Authors:** Tiziana de-Magistris, Azucena Gracia

**Affiliations:** 1 Unidad de Economía Agroalimentaria, Centro de Investigación y Tecnología Agroalimentaria de Aragón Zaragoza, Zaragoza, Spain; 2 Instituto Agroalimentario de Aragón-IA2 (CITA-Universidad de Zaragoza), Zaragoza, Spain; Middlesex University London, UNITED KINGDOM

## Abstract

The aim of this study is to test whether projection bias exists in consumers’ purchasing decisions for food products. To achieve our aim, we used a non-hypothetical experiment (i.e., experimental auction), where hungry and non-hungry participants were incentivized to reveal their willingness to pay (WTP). The results confirm the existence of projection bias when consumers made their decisions on food products. In particular, projection bias existed because currently hungry participants were willing to pay a higher price premium for cheeses than satiated ones, both in hungry and satiated future states. Moreover, participants overvalued the food product more when they were delivered in the future hungry condition than in the satiated one. Our study provides clear, quantitative and meaningful evidence of projection bias because our findings are based on economic valuation of food preferences. Indeed, the strength of this study is that findings are expressed in terms of willingness to pay which is an interpretable amount of money.

## Introduction

Empirical evidence on food choice has shown that consumers are subject to projection bias when making intertemporal decisions. In their seminal paper, [1] coined the term ‘projection bias’ to refer to a general bias which arises whenever preferences change over time, causing individuals to project their current state into the future incorrectly. In particular, when people predict future preferences, they understand the direction in which their preferences will change, but they tend to underestimate the degree to which their future preferences will resemble their current preferences [1]. Moreover, the presence of a visceral factor influence, such as hunger, is considered one of the factors that can cause projection bias [1–3].The term ‘visceral factor’ refers to a wide range of negative emotions (anger, fear, etc.) which drive states that can motivate people to engage in impulsive behaviour called ‘out of control’, characterized by unplanned and unconscious cognitive mediation [2–4]. In particular, projection bias is well illustrated when hungry people overspend in the supermarket because they tend to think that their current hunger will endure into the future. However, after having eaten a meal, they realize that if their hunger had been satisfied when they were shopping in the supermarket, they would not have overspent on these items.

The objective of this study is to provide quantified evidence of projection bias by incentivizing people to reveal their truthful preferences for different cheese products. We assessed truthful preferences using an experimental auction, asking individuals the maximum price they would pay (in other words, their willingness to pay, WTP) for various cheese products.

To our knowledge, there are at least three studies that have examined the projection bias in food choices. The first is by [5] who approached either hungry or satiated office workers, and asked them to choose between a healthy snack and a less nutritious snack (e.g. fruit vs candy bars) to be delivered one week later, either at a specific time of the day when they expected to be hungry (i.e. late in the afternoon) or when they expected to be satiated (i.e. immediately after lunch). Results of this experiment confirmed that advance choices were influenced by current hunger, because workers with anticipatedfuture hunger chose unhealthier snacks than satiated workers. On the other hand, hungry subjects chose unhealthy snacks more often for future consumption in comparison to the currently satiated subjects, leading to evidence of projection bias.

Along the same lines, [6] conducted a field experiment at a local grocery store and participants were asked to list the items they intended to purchase. The authors found that if a hungry shopper received a small piece of food right before entering the food market, they were able to limit their shopping to the initial items on their shopping list.

Finally, the study carried out by [7] approached the issue of projection bias using a home-grown experimental auction when bidding on a ham and cheese sandwich. The authors reported the existence of projection bias when subjects had to predict their future tastes versus when they bid for a product intended for immediate consumption.

Although the studies of [5][6] and [7] bear important information to the literature,they present some limitations.

Firstly, in the studies of [5] and [7], the appetite of subjects was manipulated by varying the time of day when their choices were made. Therefore, hungry and satiated states of individuals were supposed to depend on the time of day (i.e. before and after lunchtime, respectively). Consequently, those people who were invited to participate at different times of day (i.e. before and after lunchtime), might be more likely to come before lunch and others after, and then they might be influenced by a time-of-day effect.

Secondly, [5] and[6] did not provide clear, quantitative and meaningful evidence of projection bias because their findings were not based on the economic valuation of food preferences. To illustrate, the study by [5] was conducted in a hypothetical setting where participants were required to choose between a healthy and an unhealthy product. However, individuals were not asked to reveal their willingness to pay for this product. Moreover, [6] reported only differences in ‘unplanned purchases’, and ‘listless’ shoppers spent a larger proportion of their total amount on unplanned food items when they were hungry rather than when they were satiated. However, ‘lustful’ shoppers spent the same proportion of their total amount on unplanned food items both when they were hungry and when they were satiated. Hence, a limitation of the study is that the experiment design considered only the proportion of unplanned food items, thus resulting in it being complicated to connect unplanned purchases to truthful preferences.

Hence, the current study presents several strengths. Firstly, a manipulation task was applied to discriminate between initial hungry and satiated levels by feeding participants some unrelated food during the auction. Secondly, our results are expressed in terms of willingness to pay, an interpretable amount of money. Quantifying the projection bias phenomenon is quite important because of its implications for food companies and retailers when launching a new product on the food market.

Thirdly, future delivery moments were distinguished in two perceived levels of hunger or satiety of the subjects, and to attempt to have similar behaviours in a meal situation, the experiment was set up at the same time of day (before lunch at 1:00 p.m.).

Finally, in order to isolate the effect of changes in attributes and organoleptic characteristics, the study took into account a non-perishable product such as semi-cured cheese.

The findings of this study confirm that projection bias is prevalent in food decisions, since hungry subjects overvalue products delivered both in the hungry and satiated future states compared to satiated subjects. Moreover, overvaluing by currently hungry people is more pronounced when products are given in a future hungry state.

The rest of the article is organized as follows. The next section discusses the hypotheses, the experimental design, the rationale for the inclusion of the different treatments, and the steps in the implementation of the auction. The results are presented in the following section, and the final section discusses the importance and the implications of the findings.

## Materials and Methods

### Ethics Statement

Respondents were informed by written consent to participate in the study. Each participant was assigned an ID number and asked to sign a participation and ethical approval form. Protocols and procedures were approved by the Ethics Committee of the Agri-food Research and Technology Centre (CITA).

### Experimental design

We used a homegrown auction that allows for assessing consumers’ valuations for different products. This valuation method has been extensively used to value different food products in the last few years. [8] indicated that up 2006, more than 100 academic studies had utilized experimental auctions to elicit consumers’ preferences for various products. Experimental auctions are popularly used due to their incentive compatibility properties; they provide an incentive to subjects to state their true preferences.

For this experiment the random n^th^ price auction with repeated rounds (i.e. 6 rounds) and the full bidding approach were employed for several reasons. Firstly, repeated rounds have the benefit of the learning effect as stated by several authors [8,9,10] who argue that this procedure yields valuations more consistent with neoclassical economic theory. On the other hand, since we were aware of the possibility of the occurrence of bid affiliation effects and other psychological effects (such as competition or anchoring effects) no price feedback among multiple rounds was reported [11].Secondly, similar to the Becker-Degroot-Marschak (BDM) mechanism [12], the random n^th^ price works well for off-margin bidders whose values are far from the market price. However, in contrast to the BDM mechanism andas [13] reported, the random n^th^ price auction has an additional benefit of encouraging competition amongst bidders similar to the second-price auction. [14] compared the second Price auction top the BDM while [13] compared the second Price auction to the random nth. [15] compared all three mechanisms and they found that on average the second price and random nth price auctions were significally more accurate than the BDM.

We decided to use the full bidding process because it has the advantage of eliminating any aversion to loss and risk exchanging of the participants [16,17] and it is better than the endow-upgrade method when the products in the auction have the same field substitutes [18].Since in our experiment, the characteristics of our cheese products have the same field substitutes, we used the full bidding method which allowed us to eliminate the type of cheese as the third factor in our experimental design. In the full bidding, each participant had to submit simultaneously a bid for each of the auctioned products. These bids were our economic measure of interest as it is the maximum total willingness to pay (WTP) for the different cheese products. Finally, we did not use the reference price of field substitutes of cheese during the auction because the reference price of field substitutes would increase the bid values as stated by [17,19].

To avoid transaction cost effects, subjects received a signed document in which the experimenters stated that they would handdeliver the product to them the next day. To gain the trust of participants, monitors showed their personal signed business cards with a signed document.

Finally, to homogenize participants’ level of hunger in both treatments, we set the sessions before lunchtime (at 1:00 p.m.) and manipulated the hunger state during the experiment. This procedure minimized selection bias that could occur because some people might more likely participate at lunchtime, while others might more likely come after lunch.

We designed an experimental auction applying a 2x2 full factorial design. The first factor is the immediate ‘appetite level’ during the experiment (hungry vs satiated). The second factor represents ‘food attitudes’ at the moment of cheese delivery (future expected hunger at 1:00 p.m. vs future expected satiation at. 3:00 p.m). Hence, this led to the four treatment groups displayed in two rows and two columns in Table 1. The rows being H_0_ (currently hungry) and S_0_ (currently satiated) and the columns being H_1_ (future hungry) and S_1_ (future satiated). The first treatment group (H_0_H_1_) denotes that participants were currently hungry and that they would receive the cheese when they expected to be hungry. The second treatment group (S_0_H_1_) denotes that satiated individuals would receive the cheese when they expected to be hungry. The third combination (H_0_S_1_) consisted of currently hungry participants who would receive the cheese products when they expected to be satiated.

**Table 1:** Experimental design.

Level of hunger	Food attitudes	
	H_1_	S_1_
**H**_**0**_	H_0_H_1_	H_0_S_1_
**S**_**0**_	S_0_H_1_	S_0_S_1_

Finally, the fourth treatment group (S_0_S_1_) denotes that satiated participants would receive the cheese products when they expected to be satiated. Hence, projection bias is not only tested by the current levels of hunger or satiety but also by future perceived levels of hunger or satiety of the subjects, since we informed participants before the bidding process when the product would be delivered.

### Hypothesis definition

Three hypotheses were outlined to test for projection bias. The first hypothesis to test is whether the WTP for different cheese products exhibited by hungry participants is equal to the WTP for cheese products by satiated subjectswhen they will receive the cheese the next day, beingeither hungry or satiated:

$$\begin{array}{l} H0_{1}=WTP(H_{0}H_{1},H_{0}S_{1})=WTP(S_{0}H_{1},S_{0}S_{1})\\ H1_{1}=WTP(H_{0}H_{1},H_{0}S_{1})>WTP(S_{0}H_{1},S_{0}S_{1}) \end{array}$$

If the null hypothesis is rejected, it means that projection bias exists. That is because the currently hungry participants who will receive the cheese the following day when they expect to be either hungry or satiated, will presume that the current hunger will endure into the future and, thus, they will overvalue the cheeses. However, when they are currently satisfied, they will not overvalue the cheeses because of their satiated state, even if predicted to be either hungry or satiated in the future. Hence, currently hungry people underappreciate the impact of being satiated in the future on their utility, thus leading them to value the cheese more than they should have done if they were satiatedin the future.

The second hypothesis concerning projection bias is whether the WTP revealed by currently hungry or satiated participants who will receive the cheese when they are hungry is equal to the WTP for cheeses delivered the next day when they are satiated:

$$\begin{array}{l} H0_{2}=WTP(H_{0}H_{1},S_{0}H_{1})=WTP(H_{0}S_{1},S_{0}S_{1})\\ H1_{2}=WTP(H_{0}H_{1},S_{0}H_{1})>WTP(H_{0}S_{1},S_{0}S_{1}) \end{array}$$

If the null hypothesis is rejected then it means that the projection bias exists because given either state (hungry or satiated), participants were willing to pay more to get the cheese when they anticipated being hungry in the future than when they anticipated being satiated.

Finally, since there is an interaction effect between current hungry and future hungry situations and between current and future satiated situations which could exist, a third hypothesis was established. Hypothesis 3 is whether the difference in the WTP between hungry participants who will receive the cheeses when they will be either hungry or satiated are equal to the difference in WTP between satiated people who will receive the cheese when they expect to be either hungry or satiated, respectively:

$$\begin{array}{l} H0_{3}=WTP(H_{0}H_{1})-WTP(H_{0}S_{1})=WTP(S_{0}H_{1})-WTP(S_{0}S_{1})\\ H1_{3}=WTP(H_{0}H_{1})-WTP(H_{0}S_{1})>WTP(S_{0}H_{1})-WTP(S_{0}S_{1}) \end{array}$$

If the null hypotheses is rejected, it means that there is a positive difference in willingness to pay between future hungry and future satiated participants, but the projection bias makes the difference greater when one is currently hungry rather than currently satiated.

### Implementation of the auction

A non-standard subject pool consisting of 98 consumers was recruited using a stratified random procedure by age, gender, and education level. In an attempt to ensure that subjects were representative of shoppers in stores, we selected those people responsible for the food purchases in the household and who had previous experience with the products under investigation. In particular, we recruited two experimental groups;48 people participated in H_0_H_1_ and S_0_H_1_ treatments, while 50 individuals participated in H_0_S_1_ and S_0_S_1_ treatments. According to our experimental design, we were interested in recruiting hungry participants and in making them satiated during the experiment. Then, those subjects who stated that they were not hungry before being fed were excluded from the experiment, which resulted in the removal of nine participants.As shown in Table 2, the experimental groups did not differ in terms of the percentages of women and men, age, education and, income. The results of these tests suggest that our randomization was successful in equalizing the characteristics of participants between the two experimental groups.

**Table 2:** Sample characteristics and demographic variables definition %.

	Treatments			
Demographic caracteristics	H_0_H_1_	S_0_H_1_	H_0_S_1_	S_0_S_1_
**Gender**				
**Male**	50.0	50.0	40.4	40.4
**Female**	50.0	50.0	50.0	50.0
**Test (χ**^**2**^**); (p-value)**	0.8217; 0.365			
**Age**				
**Less than 35 years old**	33.3	33.3	31.0	31.0
**Between 36–54 years old**	23.8	23.8	34.0	34.0
**More than 55 years old**	42.9	42.9	34.0	34.0
**Test (χ**^**2**^**); (p-value)**	1.259;0.533			
**Education of respondent**				
**Elementary School**	14.3	14.3	19.1	19.1
**High School**	52.4	52.4	46.8	46.8
**University**	33.3	33.3	34.1	34.1
**Test (χ**^**2**^**); (p-value)**	0.453; 0.798			
**Average Household monthly income**				
**€600–€1,500**	19.1	19.1	23.4	23.4
**€1,501–€2,500**	57.1	57.1	44.7	44.7
**More than €2,500**	23.8	23.8	31.9	31.9
**Test (χ**^**2**^**); (p-value)**	1.397;0.497			

The product selected for the experiment was cheese from semi-cured pasteurized sheep milk. In particular, there were four different versions of this cheese: (i) cheese without any claim, (ii) cheese with a health-related claim (a nutritional claim indicating a fat-reduced content: ‘light’contained 40% less fat than the other cheeses in the experiment), (iii) cheese with a regional claim (‘protected designation of origin—PDO’), and (iv) cheese with an organic claim (with the European organic logo). The rest of the cheese characteristics were constant, and the only difference among them corresponded to the featuresclaimed and mentioned above.

At the beginning of the session, participants were informed that they would receive a fee of 10 euros at the end of the session. Each participant was assigned an ID number and was asked to sign a consent form. Initially, the experimenters provided the participants the instruction mechanism and the information on the products sheets. In order to avoid any communication among the participants, during the auction, each consumer was positioned separately from the others, and the monitors stressed that communication was not allowed during the auction. The subjects were fully briefed on the procedure of the auction method using a blackboard and scripts, and they were informed about the dominant strategy to reveal their true values for the products offered. Since participants were cheese consumers, they were not given any bid limits. A practice task with four different candy barswas conducted to allow participants the opportunity to understand the auction mechanism.

During the auction, each participant was asked to submit simultaneously a bid for each of the four cheese products. The bids were collected and this step was repeated during three additional rounds. At the end of the third round, we manipulated the hungry/satiated state by providing the participants with an amount of unrelated food (e.g., tortilla, vegetables, squid, tapas and water), asking them to eat until they felt satiated. Participants were not allowed to communicate with each other when eating.When all the rounds were conducted, the ‘n’ was randomly chosen to determine the binding price (n^th^ highest bid for the product). Also, a random draw determined which of the six rounds was binding. Then, a random draw determined which of the four cheese products was binding. However, the product was handdelivered to the highest bidders the next day wherever they wished. The timing of delivery on the subsequent day depended upon the treatment (i.e. before lunch or after lunch).

### Hunger rating

A manipulation check question (How hungry are you?) to measure the subjective degree of feelings of starvationwas posed to subjects before starting the auction and after having eaten the food. Responses are on a Likert scale where 1 indicates ‘not at all’ and 5 indicates the highest intensity of hunger [20–21].

## Results

### Manipulation checks

Table 3 reports the mean level of the self-reported hunger of participants and the t-test results. The averages of the self-reported hunger levels of participants before and after the manipulation of their hunger were statistically different at the 5% significance level. These results indicate that participants stated a significantly lower self-reported level of hunger after eating and that they were indeed hungry at the beginning of the experiment and became satiated afterward.

**Table 3:** Level of self-reported participants´hunger between treatments.

	Treatments			
	H_0_H_1_	S_0_H_1_	H_0_S_1_	S_0_S_1_
**Self-reported hunger level**	3.14	1.19	2.85	1.48
**t-test (p-value)**	12.2	(0.000)*	9.9	(0.000)*

*It denotes statistically significant differences at 1%, respectively

### Preliminary analysis

Table 4 reports the average bids for each cheese product across the six rounds and 4 treatments. It is observed that generally the average bids of the first round are statistically different from the second round for all cheese products (unlabelled: t = 2.81, *p*-value = 0.003; PDO: t = 2.54, *p*-value = 0.006; light: t = 2.94, *p*-value = 0.002; organic: t = 1.70, *p*-value = 0.04). However, average bids in the third round are not statistically different from average bids in the second round (unlabelled: t = 0.67, *p*-value = 0.499; PDO: t = 1.46, *p*-value = 0.146; light: t = 0.33, *p*-value = 0.739; organic: t = 0.76, *p*-value = 0.444). These results indicate that hungry participants learned and gained experience with the mechanism due to the learning effect. However, the average bids of cheese products in the fourth round, after subjects were fed, are statistically fewer than the average bids in the third round before the hunger manipulation (unlabelled: t = 1.48, *p*-value = 0.07; PDO: t = 2.04, *p*-value = 0.02; light: t = 2.49, *p*-value = 0.007; organic: t = 2.03, *p*-value = 0.002). This result implies that the hunger manipulation brought about some effect on the bidding behaviour of participants. Finally, as expected, the average bids in the fourth round are not statistically different from average bids in the fifth round (unlabelled: t = 0.51, *p*-value = 0.604; PDO: t = 1.03, *p*-value = 0.146; light: t = -0.105, *p*-value = 0.913; organic: t = 2.12, *p*-value = 0.03) and the sixth round (unlabelled: t = 0.59, *p*-value = 0.55; PDO: t = -0.28, *p*-value = 0.77; light: t = -0.12, *p*-value = 0.901; organic: t = 0.21, *p*-value = 0.827). These results confirm that individuals had already learned during the previous rounds and that hunger manipulations (current and future) were the only effects produced during the experiment.

**Table 4:** Mean bids (€/100 grams) for each cheese across treatments.

	H_0_			S_0_		
Cheese	Round1	Round2	Round3	Round4	Round5	Round6
**Unlabelled**						
H_1_	1.14	1.06	1.06	1.05	1.05	1.04
S_1_	1.07	1.02	1.00	0.94	0.92	0.92
**PDO**						
H_1_	1.46	1.38	1.38	1.32	1.32	1.31
S_1_	1.32	1.26	1.22	1.09	1.07	1.08
**Light**						
H_1_	1.35	1.28	1.28	1.19	1.21	1.20
S_1_	1.17	1.07	1.06	0.92	0.91	0.92
**Organic**						
H_1_	1.42	1.37	1.38	1.36	1.29	1.28
S_1_	1.28	1.21	1.17	1.12	1.10	1.10

### Hypothesis testing

The whole sample dataset contains the pooled bids of each subject by round and for the four cheese products. Firstly, to test the three hypotheses concerning projection bias, anANOVA analysis with a model of two main effects (H_0_ and H_1_) and one interaction between H_0_ and H_1_ was taken into consideration. Secondly, to decide the best specification for testing projection bias, we used our ANOVA model for each cheese product and for pooled cheese products. As shown in Table 5, by comparing the ANOVA models, wenoticed the existence of little evidence that the projection bias affects cheeses differently.

**Table 5:** ANOVA analysis: testing projection bias across the four cheese products and for pooled data.

	Unlabelled			PDO			Light			Organic			Pooled model		
	MS_e_	F	p-value	MS_e_	F	p-value	MS_e_	F	p-value	MS_e_	F	p-value	MS_e_	F	p-value
**H**_**0**_	0.83	4.66	0.03	2.42	8.74	0.00	2.36	6.76	0.00	0.91	2.19	0.13	6.15	19.5	0.00
**H**_**1**_	1.14	6.42	0.01	3.54	12.77	0.00	5.64	16.09	0.00	2.72	6.56	0.01	12.7	38.7	0.00
**H**_**0**_ * **H**_**1**_	0.17	0.98	0.32	0.27	0.98	0.32	0.24	0.70	0.40	0.03	0.08	0.77	0.65	2.08	0.15

This result was also confirmed by the ANOVA analysis using the type of cheese as third factor and the covariance analysis using the Likelihood Ratio (LR).

Hence, to test the hypotheses of projection bias we considered the pooled model consisting of average bids for the four cheeses as shown in the last columns of Table 5. Moreover, the average pooled bid values across the four treatments are shown in Table 6.

**Table 6:** Average Bids across the four treatments(€/100 grams).

Treatments	Mean bids	Standar Deviation
**H**_**0**_**H**_**1**_	1.29	0.61
**S**_**0**_**H**_**1**_	1.22	0.56
**H**_**0**_**S**_**1**_	1.15	0.54
**S**_**0**_**S**_**1**_	1.00	0.52

Generally, as shown in Table 6, the average bids for all cheeses were higher when subjects were currently hungry than when they were satiated. To illustrate, the average bids for cheese products were higher in H_0_H_1_ than in S_0_H_1_. Moreover, average bids were higher in H_0_S_1_ than in S_0_S_1._These results are confirmed by the ANOVA analysis as illustrated in Table 4. It is evident that the main effect of current hunger H_0_was on average bid values, meaning that we are able to reject the first hypothesis of projection bias. This result suggests that participants overvalued the cheese products more when they were hungry rather than when they were satiated, both when they would expect to receive the cheese when they would be either hungry and satiated. Hence, for any given future state, subjects were willing to pay more when they were currently hungry than when they were satiated.

On the other hand, the average bids in H_0_H_1_ were higher than in H_0_S_1_. Likewise, the average bids in the S_0_H_1_ were higher than in S_0_S_1_,thus suggesting that participants overvalued the cheeses more when they were delivered in the future hungry treatment than in the satiated one. Indeed, the ANOVA analysis also confirmed the main effect of future hunger (H_1_) on average bid values, suggesting that we are able to reject the second hypothesis of projection bias. This result implies that for any current hungry or satiated state, participants were willing to pay more when they anticipated being hungry. Finally, the absence of significant interaction between H_0_ and H_1_suggests that we are not able to reject the third hypothesis. From the results, there was no statistical basis for concluding that immediate hunger level of individuals had a statistically greater effect on average bid values in comparison to future hunger levels, and that the projection bias does not make the difference between H_1_ and S_1_ when one is currently hungry rather than currently satiated. In other words, current hunger,H_0,_and future hunger,H_1,_had an independent and added effect on average bid values since the empathy gaps were of about equal size as stated by [5].

In conclusion, overall findings suggest clear, quantifiable evidence of the projection bias in consumer food preferences. In particular, currently hungry participants overvalued cheese delivered in the future treatments with an additional premium of 11 cents on average, compared to satiated subjects. Overvaluing by currently hungry participants was slightly more pronounced (12.5 cents) when cheese was delivered in a future hungry treatment rather than in the satiated one.

## Conclusions

It is widely documented in experimental settings from social psychology literature that individuals can often make decisions that are not in their best interest (e.g. in terms of their long-term health). One of the reasons is that the level of hunger of individuals could make difficult to predict their behaviour when they will be in a different emotionally ‘cold’ state (e.g. satiated) because their current ‘hot’ state (e.g. hungry) overrides them. Therefore, while individuals understand the directions in which their tastes will change, they can be prone to systematically underestimate or overestimate the magnitude of these changes.

The objective of our study was to test the existence of projection bias using a non-hypothetical artefactual experiment (i.e. experimental auction) to provide quantified evidence of projection bias. Specifically, we examined whether projection bias exists when hungry and non-hungry subjects were incentivized to reveal their WTP. Participants were fed to the point of satiety after a few rounds of the auction to manipulate their hunger level.

Results confirmed the existence of projection bias when consumers made their decisions concerning food products. Specifically, projection bias exists because, for any future state, participants were willing to pay a higher price premium for the cheese when they were currently hungry than when satiated. In addition, projection bias exists because given any current state (hungry or satiated), participants were willing to pay more to get the cheese when they anticipated being hungry in the future. However, we are not able to conclude whether an immediate hunger level had a statistically greater effect on average bid values than a future hunger level.

The existence of projection bias in our results has marketing implications for the food industry. Firstly, food companies are advertising their food products indeed when consumers are expected to be hungry when exposure to food advertising might create expectations in terms of tastier food. Because food advertising rises emotional utility for consumers theymay perceive food productsas worth more than their objective value, and so they are willing to pay more to get them. Secondly, food advertising could stimulates the desire for a product not only when people are currently hungry, but also when they anticipate being hungry because the palatable food stimuli can trigger an hedonic hunger. Finally, another possible marketing strategy conducted by food companies is to set testing promotions at stores and supermarkets before lunch and dinner time. Therefore, shoppers might start to crave the food and then to project how much food they want or need, thereby driving up sales, whether or not they were initially hungry.

However, from the consumer`s perspective, targeting hunger may drive consumers to purchase unhealthy food such as junk food because they become more sensitive to future cravings when they are currently experiencing a craving. Therefore, the findings of this study also suggest that individuals should avoid buying food when they are in a hungry state because they will be more likely to purchase unhealthy food and pay more for it than when they are satiated.

Finally, our results also have significant implications for the design and use of non-hypothetical experimental auctions to elicit WTP values from subjects for food products. For example, our results suggest that if participants have projection bias, their bidding decisions can be influenced by their current levels of hunger or satiety. This finding is important since hunger levels of participants are not taken into account in experimental auctions. Given the increasing importance and use of experimental auctions to elicit consumers’ preferences and WTP values for marketing and policy purposes, researchers and practitioners should take into account the hunger level and control this factor when designing an experimental auction. If this is not taken into account, valuations may be skewed by participants’ projection bias.
